# E-bibliotherapy for improving the psychological well-being of informal caregivers of people with dementia: a randomized controlled trial protocol

**DOI:** 10.1186/s12912-024-01706-5

**Published:** 2024-02-01

**Authors:** Shanshan Wang, Jing Qin, Daphne Sze Ki Cheung, Stefanos Tyrovolas, Sze Him Isaac Leung, Angela Yee Man Leung, Patricia Mary Davidson

**Affiliations:** 1 School of Nursing, The Hong Kong Polytechnic University, Hong Kong SAR, China; 2 Department of Statistics, The Chinese University of Hong Kong, Hong Kong SAR, China; 3 University of Wollongong, Wollongong, NSW Australia; 4 Department of Nutrition and Food Studies, George Mason University, Fairfax, VA USA

**Keywords:** Bibliotherapy, Electronic, Dementia, Caregiver, Psychological well-being, Nursing

## Abstract

**Background:**

Providing informal care for individuals with dementia is frequently a challenging and demanding experience that can have detrimental effects on the psychological well-being of caregivers. Regrettably, community-based caregiver services often prove inadequate, highlighting the necessity for innovative approaches to support caregivers.

**Aim:**

To test the efficacy of e-bibliotherapy in improving the psychological well-being of informal caregivers of people with dementia.

**Method:**

The study is divided into two phases. In phase 1, the research team will co-design the e-bibliotherapy app with caregivers. In phase 2, a randomized controlled trial will be conducted among 192 informal caregivers of people with dementia in Hong Kong. Caregivers will be randomly assigned to either the e-bibliotherapy group or the control group using simple randomization. Outcome measures will encompass caregivers’ psychological well-being, caregiving appraisal, mental health, saliva cortisol levels as an indicator of stress, and health-related quality of life for caregivers. Data will be collected at baseline, immediately post intervention, and 3 months and 6 months post intervention. General linear mixed model will be employed to analyze intervention effects. Qualitative interviews will be undertaken to explore caregiver experiences within this study and evaluate intervention acceptability using conventional content analysis methods.

**Discussion:**

This study represents a pioneering effort in utilizing e-bibliotherapy to enhance the psychological well-being of informal caregivers of individuals with dementia, addressing the existing gap in caregiver services and facilitating knowledge dissemination within the community.

**Trial registration:**

The trial has been registered on ClinicalTrial.gov (Ref: NCT05927805).

## Background

As the global population ages, the prevalence and burden of dementia are escalating, particularly among older adults [1]. In 2019, over 55.2 million people worldwide were living with dementia, and projections indicate that this number will rise to 78 million by 2030 and 139 million by 2050 [2]. Consequently, this condition necessitates an enhancement of services for people with dementia by governments and healthcare professionals. However, the availability of services remains insufficient to meet the growing demand [3]. As a result, informal caregivers assume a crucial role in providing primary care to individuals with dementia [4].

Approximately 85% of older adults with mild to moderately severe dementia receive care from informal caregivers [5], who are unpaid unprofessional caregivers such as family members [6]. However, caring for a person with dementia can be demanding due to the complex behavioral and psychological symptoms, stigma, and safety risks, which can have detrimental effects on caregivers’ psychological well-being, quality of life, and lead to stress, anxiety, and depression [7–9]. More than 80% of caregivers report high-level stress, and nearly half experience depression during their caregiving role [10]. The poor psychological state of informal caregivers can result in inadequate care and early placement in nursing homes for people with dementia [11]. Our survey of Chinese dementia caregivers revealed that their psychological well-being was unsatisfactory, characterized by low autonomy and low environmental mastery. This was particularly evident during the COVID-19 pandemic, with 72.1% of caregivers experiencing clinical or major depression, in addition to high levels of stress [12]. Another study found that approximately 80% of working dementia caregivers felt depressed or hopeless, and 60.8% reported poor or fair health status [13].

Given the deleterious effects on psychological well-being, informal caregivers have articulated the need for health and social services [14]. The majority of informal caregivers have emphasized their need for assistance with day-to-day care and striking a balance their caregiving role and personal needs [15]. At present, various counselling, emotional support, and respite services are provided for caregivers in Hong Kong [16]. However, the utilization rate of dementia caregiver services remains generally low (< 40%), typifying a global phenomenon [17].

Several factors contribute to this phenomenon. Firstly, the current caregiver services are nonstructured and primarily consist of generic counseling that does not cater to the specific needs of caregivers [16]. Secondly, the limited availability of healthcare professionals results in extensive waiting lists and poor accessibility, with the majority of applicants unable to receive services even after half a year of waiting [16]. Thirdly, caregivers often hesitate to seek help due to their high workload, emotional over-involvement, and a strong sense of filial or social obligation [18]. Our interviews revealed that Chinese dementia caregivers exhibit significant tolerance and reluctance to use services, stemming from their fear of not fulfilling their filial duties [19]. Lastly, and more fundamentally, it is unsustainable to solely rely on external services to address health problems caused by long-term care, and more fundamental changes may be necessary to support informal caregivers.

In light of the current situation, a multitude of studies have been conducted. A published systematic review indicates that psychosocial and psychoeducational interventions tend to be beneficial to dementia caregivers [20]. Among these interventions, cognitive-behavioral techniques have been found to be the most beneficial for enhancing caregiver mental health [21]. Despite the therapeutic benefits demonstrated in previous studies, current services and interventions are predominantly provided in face-to-face or group-based formats, which may not be feasible for those encountering challenges in securing substitute caregivers or those who hesitate to seek help due to stigma [22]. Consequently, there is a pressing need to implement accessible, feasible, convenient, and sustainable interventions that can be integrated into community services. A systematic review revealed that individual self-help interventions could aid in overcoming existing barriers and are more effective than group interventions [23]. It is crucial to provide interventions that can be tailored to the specific needs of caregivers, delivered in a format that is accessible to all caregivers, and sustained over the long term.

Bibliotherapy, an innovative non-pharmacological self-help intervention, has the potential to address the criteria mentioned above. Bibliotherapy is an individual intervention that involves reading materials for therapeutic benefits [19]. Our systematic review indicates that bibliotherapy is effective in improving mental health, with sustained effects [6]. This model of intervention that applies cognitive-behavioral techniques requires few resources and has good sustainability, as caregivers can continue using the reading materials even after the intervention period. The mechanism of bibliotherapy is typically based on the process of the ‘identification-catharsis-insight” process, which guides caregivers to identify their problems, achieve emotional release, and gain insight into cognitive reframing [19].

Our pilot study demonstrated the feasibility and preliminary efficacy of bibliotherapy in enhancing caregivers’ psychological well-being and caregiving appraisal [19]. However, several factors necessitate the need for a full trial. To begin with, participants from the pilot study suggested modifications to the intervention protocol to better cater to caregivers’ needs and enhance usability. The conventional delivery method of written materials restricts dissemination to those with low literacy levels. To overcome this, participants proposed an e-bibliotherapy approach, such as a user-friendly app, to facilitate dissemination to a broader range of caregivers, regardless of their educational background [19]. Our review corroborated the effectiveness of e-bibliotherapy in improving the mental well-being of dementia caregivers [6]. Moreover, although the effect size of caregiving appraisal was moderate, the effect size on psychological well-being was small in the pilot trial. Given that caregiving appraisal is the mechanism driving changes in psychological well-being [12], the small-scale pilot study might have been underpowered to detect a genuine effect on psychological well-being. To validate the effect size observed in the pilot study and establish the generalizability of the findings, a trial with a larger sample size is still required. Furthermore, the pilot study only examined the immediate post-intervention effect, while evidence suggests a sustained effect of bibliotherapy, which has not been extensively studied [6]. Therefore, in this study, we will develop and test an e-bibliotherapy app-based intervention within a fully powered randomized controlled trial. This implementation of this intervention has the potential to facilitate an accessible, convenient, and sustainable approach to enhance the psychological well-being of informal caregivers of people with dementia, ultimately leading to an improvement in the quality of care provided to those with dementia.

## The study

### Aims

To evaluate the efficacy of e-bibliotherapy in improving the psychological well-being of informal caregivers of people with dementia.

### Hypothesis

Participants in the e-bibliotherapy group will demonstrate a greater improvement in psychological well-being than the control group immediately post intervention, and three and six months after completing the intervention.

## Methods

### PHASE I co-design an E-bibliotherapy app

Informed by the feedback obtained from our pilot study and literature review, an e-bibliotherapy app is being developed. The app’s design follows senior-friendly website guidelines, such as font size, contrast, button style and size, menus, and navigation, to ensure usability for caregivers, who are mainly middle-aged and older adults.

To protect the privacy of the participants, each participant will be given a project ID and password to log in to the app. No personal information will be collected while using the app. Only authorized participants and research team members can log in to the app and access the research data. These measures will ensure that the participants’ privacy is protected and that the data collected are secure.

#### App functionalities

The E-bibliotherapy App will include several functions to support the users. The learning functionality will be used to upload the contents of the bibliotherapy manual that was tested in the pilot study. The manual comprises eight chapters, each of which is divided into several sub-sessions. Explanatory mini videos highlighting key information from each session will be produced to enhance participants’ comprehension. Audiobook functionality will be used to allow users to listen to the materials. Mandatory task functionality will be set up to facilitate establishing designated compulsory tasks. Participants will be restricted from advancing to the next session until they have completed the requisite mandatory tasks. Voice input functionality will be used to allow users to complete tasks via auditory input. An e-bulletin will be set up to enable users to read the latest updates and reminders. Finally, a messenger function will allow users to communicate with the researchers. In addition to the above-mentioned functions for users, there will also be a statistical functionality for researchers to monitor the learning progress of each participant and calculate the task completion rate automatically.

#### Co-design the E-bibliotherapy app with end-users

To ensure that the E-bibliotherapy App meets end user’s needs, we will invite them to co-design the app through focus group interviews. The feedback collected from the interviews will be used to maximize the app’s feasibility, usability, and acceptability. Below are the details of the co-design:

##### Sampling

Purposive sampling will be used to recruit a relatively diverse sample in terms of age, gender, educational level, caregiving duration, caregiving intensity, etc. We plan to invite 4 to 8 caregivers in each focus group. Thematic saturation will be used as the criterion for ceasing the interviews.

##### Eligibility criteria for caregivers

To participate in the study, caregivers must meet the following inclusion criteria: (1) be primary caregivers aged 18 or above; (2) provide unpaid regular care to a person with mild or moderately severe dementia consistent with a score between 4 and 6 using the Global Deterioration Scale [24]; (3) have cared for the care recipient for at least six months; (4) assist with at least one of the care recipient’s daily activities; (5) use a smartphone or tablet; and (6) be able to read Chinese.

Caregivers will be excluded if they (1) have unstable physical or mental conditions; (2) have cognitive impairment; (3) are undergoing acute treatment or have not yet stabilized on their chronic medication; and (4) are involved in another interventional study.

##### Data collection

To ensure that participants are familiar with the E-bibliotherapy App prototype, they will be asked to attend a briefing session two days before the focus group. During the briefing, the app prototype will be installed on their smartphones or tablets, allowing them adequate time to acquaint themselves with the app’s features and functionalities.

Each focus group will include a moderator and a note-taker to ensure that all participants’ feedback is captured accurately. Open-ended questions will be asked (e.g., *In general, what do you think of the prototype? What do you think is good about the app? What difficulties or barriers have you encountered when using the app? What recommendations do you have to make the app better fit your needs?*). The interviews will be audio-taped under the participants’ consent. Each interview will last for 60 to 90 min, depending on the natural break. Conventional content analysis will be conducted to capture the feedback from the focus group interviews.

##### E-bibliotherapy app debugging

The app will be adjusted according to the co-design findings and go through pilot testing among 12 caregivers to test the app’s usability [25]. Then, the app will be further debugged based on the pilot study comments.

### PHASE II randomized controlled trial

#### Study design

A single-blinded, parallel-group, two-arm randomized controlled trial. The study protocol complies with the SPIRIT checklist [26] and is registered at ClinicalTrial.gov (Ref: NCT05927805).

#### Setting and sampling

Convenience sampling will be used to recruit participants from government-subsidized community-based elderly service centers. Staff from the centers will recommend potential participants to the research team, and a research assistant will assess the eligibility against the criteria mentioned in the co-design stage. The recruitment process is anticipated to commence in May 2024.

#### Sample size

The sample size was calculated by testing between-and-within interactions in a General Linear Mixed Model (GLMM), using the software “General Linear Mixed Model Power and Sample Size”. Type I and II errors were set at 0.05 and 0.2, respectively. The marginal means of psychological well-being from the pilot study at baseline and post intervention were 84.07 and 86.35, respectively, for the intervention group. For the control group, they were 73.5 and 70.7, respectively. We assume that the follow-up values for the intervention group are equal to 85.7 and 85.2; 71 and 71.3 for the control group. The standard deviation of the pilot study was 13. An autoregressive correlation structure (AR1) with a correlation equal to 0.7 was used. To achieve alpha = 0.05 and power = 0.801, the estimated sample size would be 160. Considering the 20% attrition rate of the pilot study, the estimated sample size would be 192 in total.

#### The intervention group

Participants in the intervention group will be provided with access to the e-bibliotherapy app installed on their smartphones. A standardized bibliotherapy manual will also be distributed to them for convenient reference. The manual, which has been pilot tested, comprises eight chapters, each approximately ten pages in length [19]. Each chapter centers on a single active component that could potentially influence a caregiver’s psychological well-being. The intervention materials from the manual (texts, relevant explanatory images, and assignments), matched with corresponding videos and audio, will be uploaded to the app, and only the intervention group will be granted authorization to access them.

During the intervention, participants will undergo eight weekly sessions of e-bibliotherapy (Table 1). Each session will entail viewing an explanatory video, reading a chapter or listening to the audiobook, and completing the corresponding tasks. This is an individual self-help intervention, enabling caregivers to complete the watching and reading at their convenience, at any time and location. Telephone coaching, if required, can also be availed. The research team will monitor participants’ intervention completion levels through statistical functionality. For those who have completed less than 60% of the tasks or require assistance with problems, standardized protocol-based telephone coaching will be arranged. The coaching aims to enhance participants’ adherence and safety, and will be conducted by a trained research assistant with a background in psychology or nursing. Each coaching session will last for no more than 30 min, as reported by our pilot study. The coaching manual encompasses troubleshooting plans, such as behavior management, caregiver distress, and caregiver discouragement, which are designed to address unforeseen circumstances [19].

**Table 1:** Intervention protocol for this study

Weekly tasks	Session	Contents
Watch session 1 videos Read session 1/listen to audiobook session 1 Finish assignment/coaching	Dementia and caregiver health	• What is dementia • Stages and symptoms of dementia
		• Can dementia be cured
		• How providing care can affect you as a caregiver
Watch session 2 videos Read session 2/listen to audiobook session 2 Finish assignment/coaching	Controlling care recipient behavioral problems	• Learning more about behavioral problems
		• Finding the “triggers” for problem behaviors
		• Ways to change care recipient behavioral problems
Watch session 3 videos Read session 3/listen to audiobook session 3 Finish assignment/coaching	Home safety and daily caregiving skills	• How to ensure home safety
		• How to deal with difficulties in daily care
		• Some financial and legal issues in caregiving
Watch session 4 videos Read session 4/listen to audiobook session 4 Finish assignment/coaching	Improving the dyadic relationship	• How to communicate with the care recipient
		• Using non-verbal communication to improve the relationship
		• Increasing pleasant events with the care recipient
Watch session 5 videos Read session 5/listen to audiobook session 5 Finish assignment/coaching	Improving confidence	• The importance of confidence in caregiving
		• How to improve caregiving confidence
		• Some “Basic Rights” of caregivers
Watch session 6 videos Read session 6/listen to audiobook session 6 Finish assignment/coaching	Recognizing and relieving stress	• Danger signals and how to recognize early signs of stress
		• Skills of relaxation and why it is so important for caregivers
		• Using relaxation in stressful caregiving situations
Watch session 7 videos Read session 7/listen to audiobook session 7 Finish assignment/coaching	Recognizing and dealing with depression	• Recognizing common symptoms of depression
		• Depression and its influence
		• How some little daily events can help prevent or reduce depression
Watch session 8 videos Read session 8/listen to audiobook session 8 Finish assignment/coaching	Improving family coping and seeking social support	• Family coping in dementia caregiving
		• Ways to improve family coping
		• How to seek help from relatives and friends
		• How to seek help from professionals

#### The control group

Participants in the control group will utilize the identical e-bibliotherapy application, yet they will only be authorized to access another portal offering general daily living knowledge distinct from the intervention content. They will be requested to complete a session weekly for a duration of eight weeks. Upon project completion, they will gain access to the intervention group’s content.

#### Outcomes

##### Psychological well-being

Psychological well-being will be measured with the Chinese version of Ryff’s Psychological Well-being Scale [27]. It is a 6-point Likert scale with 1 = *strongly disagree* to 6 = *totally agree*. A higher score indicates better psychological well-being. It includes 18 items and is divided into 6 subscales: positive relations with others, autonomy, environmental mastery, personal growth, purpose in life, and self-acceptance. The Cronbach’s α of the whole scale is 0.936, and the αs for the subscales ranged from 0.709 to 0.910 in our pilot study [19].

##### Caregiving appraisal

The Chinese version of the Caregiving Appraisal Scale [28] will be used. It is a 5-point Likert scale, with higher scores indicating more positive caregiving appraisals. It includes 26 items and is divided into 4 subscales: caregiving burden, caregiving satisfaction, caregiving mastery, and caregiving impact. The Cronbach’s α of the whole scale was 0.883, and the αs for the subscales ranged from 0.651 to 0.854 in our pilot study [19].

##### Mental health

The Chinese version of the Depression Anxiety Stress Scale-21 (DASS-21) will be used to measure the mental health problems of the participants. The DASS-21 is a 4-point Likert scale. It includes 21 items with three dimensions: depression, anxiety, and stress. The DASS-21 has demonstrated good reliability among Chinese adults, with Cronbach’s α ranging from 0.80 to 0.83 for each subscale [29].

##### Biomarker of stress

Saliva cortisol concentration will serve as a stress biomarker, as cortisol is the major hormone that reflects stress levels and helps the body cope with stress. It is a sensitive measure of stress among dementia caregivers [30], with morning cortisol levels validly predicting daily stress levels. Therefore, saliva samples will be collected upon waking for cortisol assessment.

##### Health-related quality of life

The 12-item Short Form Survey (SF-12) will be used [31]. The SF-12 is often used to indicate quality of life and as a proxy for health status. According to Lam et al. (2013), the SF-12 represents a valid, reliable, and sensitive measurement among the Hong Kong Chinese population. The Cronbach’s α of the scale is 0.7.

##### Covariates

Demographic data about various aspects of the caregiving situation will be collected at baseline, such as age, gender, kinship, education level, caregiving duration, intensity of care, and the care recipient’s stage of dementia.

#### Participant timeline

**Fig. 1:**
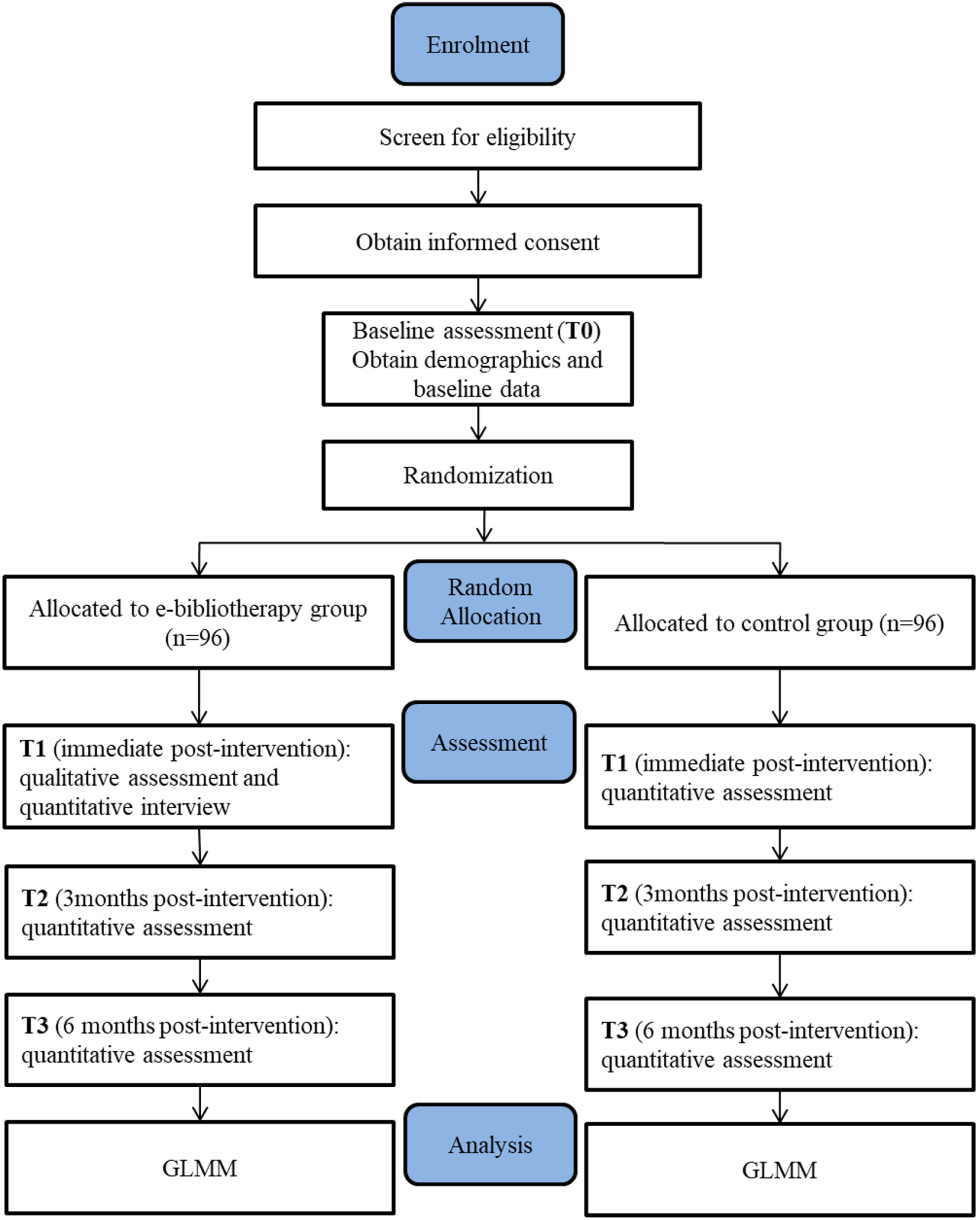
The CONSORT flowchart

The study flowchart is shown in Fig. 1, and the study timeline is shown in Table 2. Participants will be assessed at four time points: baseline (T0), immediately post intervention (T1), 3 months (T2), and six months (T3) post intervention. After receiving consent from the participants, a research assistant who is blinded to the group allocation will conduct the baseline assessment (T0). Each participant will be coded with a subject ID number that is not related to their personal information. Qualtrics will be used to collect survey data. Telephone interview will be used as a contingency plan in case the caregiver requests help in filling out the questionnaires.

Before project initiation, a briefing session will be conducted to train participants on how to use the E-bibliotherapy App and collect and store saliva samples to assess a stress biomarker. This is because the stress response is considered a potential mechanism that can negatively impact psychological well-being [32]. A Salivette® collection device kit (Sarstedt, Nümbrecht, Germany) and a user guide will be distributed to each participant.

**Table 2:** Time schedule of enrolment, interventions, and assessments

	Study Period				
	Enrolment	Allocation	Post-allocation		
Timepoint	-t_0_	t_0_	t_1_	t_2_	t_3_
	***1–9 m***	***1–9 m***	***4–12 m***	***7–15 m***	***10–18 m***
**Enrolment**:					
Eligibility screen	X				
Informed consent	X				
Allocation		X			
**Interventions**:					
E-bibliotherapy group		There is an arrow covering T0 to T2			
Control group		There is an arrow covering T0 to T2			
**Assessments**:					
Psychological well-being (Ryff’s psychological well-being scale)		X	X	X	X
Caregiving appraisal (Caregiving appraisal scale)		X	X	X	X
Mental health (DASS-21)		X	X	X	X
Biomarker of stress (Saliva cortisol concentration)		X	X	X	X
Health related quality of life (SF-12)		X	X	X	X

Note: t_0_: baseline assessment, t_1_: immediately post-intervention, t_2_: 3 months post-intervention, t_3_: 6 months post-intervention

#### Randomization, blinding, and allocation concealment

Stratified randomization will be used to allocate the participants within two weeks after the baseline assessment. Firstly, caregivers will be grouped into two strata, one comprising adult child caregivers and the other consisting of spousal caregivers. In order to ensure a balanced number of different types of caregivers in each group, simple randomization will then be performed with each stratum. The allocation sequence will be generated by the statistician on our research team, and a research assistant who is not involved in data collection will assign participants to their respective groups. The sequence of the participants will remain concealed until group allocation is designated. The data collector and community healthcare professionals will be blinded to the group allocation. Given the nature of the intervention, it will be impossible to blind the participants and the intervention facilitator.

#### Nested qualitative study at T1

A nested qualitative study will be conducted at T1 to explore participants’ experiences using the intervention and facilitate knowledge translation. Focus group interviews will be conducted with participants representing varying intervention completion levels. The interview guide will encompass overall experiences with the intervention, perceived benefits, facilitators, encountered barriers, and suggestions for improvement. Thematic saturation will be used as the criterion for ceasing the data collection.

#### Data analysis

##### Quantitative data analysis

R software will be used for analyses. Descriptive statistics will be used to summarize the baseline data. Independent t-test, Mann-Whitney U test, or chi-square test will be used to compare the baseline data. To evaluate the intervention’s efficacy, GLMM will be used. In all analyses, a significance level of 5% will be used. Cortisol concentrations will be analyzed at a collaborating laboratory using radioimmunoassay.

##### Qualitative data analysis

The recordings of the focus groups will be transcribed within 24 h, and two researchers will organize and code the transcripts using NVivo 12.0 software (QRS International, Doncaster, Australia). Conventional content analysis with an inductive approach will be performed by constantly comparing the transcripts to generate codes [33]. A codebook will be developed after analyzing the responses from the first focus group. If there are any discrepancies in coding, the two coders will discuss them and consult with a third researcher, if necessary, until a consensus is reached. The codes will then be developed into subcategories and consolidated into categories. To ensure the credibility of the content analysis, a member check will be conducted.

#### Intervention fidelity

The intervention is conceptually derived using an evidence-based approach [6, 19]. To standardize intervention quality, the intervention will be delivered via standard videos, audio, and texts. Caregiver completion of the intervention will be monitored via app statistics, and treatment skill enactment will be assessed through statistical functionality and weekly assignments. Coaching sessions will follow a manual and be audio recorded for fidelity checking. Borrelli’s tool [34] will be used to assess the fidelity of the study. A steering committee with three experts in gerontology and dementia research practice was formed to monitor the research process and data management. The research team will report to the steering committee annually for the research progress.

## Discussion

This study aims to enhance the psychological well-being of informal caregivers of people with dementia through an accessible, easy-to-use, and sustainable intervention that overcomes caregivers’ barriers to participating in psychosocial interventions. The e-bibliotherapy protocol is developed based on solid evidence, including a series of systematic reviews, surveys among informal caregivers of people with dementia, and a pilot study [6, 12, 17, 19, 35]. Our program aligns with the imperative of implementing interventions that address the critical problems among dementia caregivers identified by other publications, and the intervention components cover different emergent needs of caregivers [16, 19]. Efficacy in improving caregiver psychological well-being is expected with sustained effects. As it is a self-help program that does not involve much manpower compared to current caregiver services, it can lower the economic burden on the healthcare system.

An innovative aspect of this project is facilitating dementia caregivers to engage in self-help through an accessible and economical approach. Our study differs from current services as we focus on the positive aspects of caregivers’ psychological well-being, such as self-acceptance, meaning in life, and personal growth and development, which require urgent attention. Current non-pharmacological interventions typically focus on reducing caregiver distress and burden [36]. However, the caregiving experience is multi-dimensional, and the positive aspects cannot be ignored as they can offset adverse health outcomes [37]. Improving the psychological well-being of informal caregivers of people with dementia may help sustain them in the caregiving team and promote ageing in place.

To the best of our knowledge, this is the first study to provide bibliotherapy through an app tailored for dementia caregivers. Unlike existing commercial apps, this project will design an e-bibliotherapy app through co-design with end-users and utilize evidence-based approaches to ensure that the app’s functions are suitable for dementia caregivers. The intervention contents uploaded to the app will be based on the bibliotherapy manual, which was written using a generic approach. Only minor modifications are needed to tailor it to the specific cultural context, making it potentially applicable in other counties and settings.

Furthermore, we will use both qualitative and quantitative methodologies to analyze the effects and impact of the intervention. Quantitative scales and biomarkers will be used to measure the outcomes of interest. Moreover, a nested qualitative study will be conducted to further investigate caregivers’ experiences in the program, allowing researchers to disseminate knowledge after the study. By applying a multi-method design, we expect to gain a comprehensive understanding of implementing an e-bibliotherapy program. The integration of quantitative and qualitative data will ensure a more robust validation of this project.

## Data Availability

Not applicable.
